# The Blood–Brain Barrier as an Integration Hub in Alzheimer's Disease: How Microbiota Metabolites Modulate Central Signal Processing

**DOI:** 10.1002/cns.70703

**Published:** 2025-12-14

**Authors:** O. Giangiulio, R. Maccarone

**Affiliations:** ^1^ Department of Biotechnological and Applied Clinical Sciences University of L'aquila L'aquilla Italy

**Keywords:** Alzheimer's disease, blood–brain barrier, gut–brain axis, integration hub, lipopolysaccharide, microbiota metabolites, short‐chain fatty acids, signal processing, trimethylamine N‐oxide

## Abstract

**Background:**

While both gut–brain axis dysfunction and blood–brain barrier (BBB) breakdown are documented in Alzheimer's disease (AD), current research treats these as separate phenomena. However, emerging evidence suggests that the BBB may function as an active integration interface that processes microbiota‐derived metabolites and thereby potentially modulates how peripheral signals influence cognitive health.

**Objective:**

This review synthesizes current evidence on microbiota metabolites as modulators of BBB integration capacity, discussing how such mechanisms may contribute to variability in cognitive outcomes despite similar gut microbiome profiles by demonstrating how BBB signal‐integration mechanisms determine gut–brain communication effectiveness in AD.

**Methods:**

We analyzed peer‐reviewed literature from 2010 to 2025, focusing on BBB dynamic properties, microbiota metabolite effects on BBB function, and their integration patterns, emphasizing functional evidence supporting the BBB's active signal processing capabilities.

**Results:**

Current evidence suggests that the BBB exhibits integration properties, including dynamic permeability regulation, context‐dependent metabolite processing, and coordinated responses to complex signal streams. Short‐chain fatty acids enhance integration capacity through HDAC inhibition and coordinated receptor activation, while lipopolysaccharides and trimethylamine N‐oxide may overwhelm integration processes through TLR4‐mediated disruption. BBB dysfunction precedes classical AD pathology and correlates with altered metabolite processing capacity. Individual variations in BBB integration capacity may help account for why individuals with similar gut microbiome profiles show different cognitive outcomes.

**Conclusion:**

Viewing the BBB as an active integration interface offers a useful perspective for organizing current evidence on gut–brain interactions in AD. This conceptual perspective suggests that therapeutic strategies might benefit from supporting BBB integration capacity and optimizing metabolite‐processing mechanisms alongside improving gut health.

## Graphical abstract

The review reframes the blood–brain barrier as an active integrator of microbiota‐derived signals that shape vulnerability to Alzheimer's disease. Viewing the BBB as a dynamic processing interface between gut and brain clarifies why identical metabolite patterns yield different outcomes and opens new opportunities for earlier diagnosis and targeted therapeutic strategies.

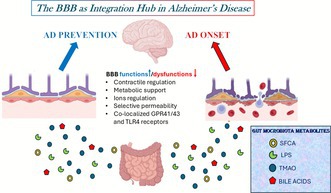

**Abbreviations**
AD: alzheimer's disease
AhR: aryl hydrocarbon receptor
BBB: blood–brain barrier
cAMP: cyclic adenosine monophosphate
CD14: cluster of differentiation 14
CI: confidence interval
CSF: cerebrospinal fluid
FMO3: flavin‐containing monooxygenase 3
FXR: farnesoid X receptor
GLP‐1: glucagon‐like peptide‐1
GLUT1: glucose transporter 1
GPR41/43: G‐protein–coupled receptors 41/43
HDAC: histone deacetylase
HR: hazard ratio
IL‐1β: interleukin‐1 beta
IL‐6: interleukin‐6
IL‐10: interleukin‐10
LAT1: L‐type amino acid transporter 1
LPS: lipopolysaccharide
LRP1: low‐density lipoprotein receptor‐related protein 1
MCT1/2: monocarboxylate transporters 1/2
MD‐2: myeloid differentiation factor 2
MyD88: myeloid differentiation primary response 88
NF‐κB: nuclear factor kappa B
PDGFRβ: platelet‐derived growth factor receptor β
P‐glycoprotein: P‐glycoprotein efflux pump
PET: positron emission tomography
PXR: pregnane X receptor
ROS: reactive oxygen species
SCFA: short‐chain fatty acids
sPDGFRβ: soluble platelet‐derived growth factor receptor β
TGF‐β: transforming growth factor β
TGR5: takeda G‐protein‐coupled receptor 5
TLR4: toll‐like receptor 4
TMA: trimethylamine
TMAO: trimethylamine N‐oxide
TNF‐α: tumor necrosis factor α
VEGF‐α: vascular endothelial growth factor α
ZO‐1: zonula occludens‐1

## 1 Introduction: The Blood–Brain Barrier Integration Hub Paradigm

Current Alzheimer's disease research has identified compelling associations between gut health and cognitive function, yet these relationships remain inconsistent and poorly understood mechanistically. While both gut–brain axis dysfunction and blood–brain barrier (BBB) breakdown are well documented in AD, these phenomena have often been studied in isolation, limiting the understanding of how they interact to influence disease progression.

Here we outline a conceptual framework in which the BBB acts as an active integration hub that processes microbiota‐derived metabolic and inflammatory signals. In this view, BBB integration capacity shapes whether peripheral inputs support or compromise cognitive function, offering a unifying perspective that may explain individual variability in gut–brain communication outcomes.

To clarify how these peripheral and central mechanisms intersect, the following sections summarize key evidence supporting active, bidirectional communication across the gut–BBB–brain axis.

### 1.1 Evidence for Active Integration Processing

The BBB demonstrates sophisticated signal‐processing capabilities that distinguish it from passive filtration barriers. Iadecola [1] provided groundbreaking evidence using two‐photon microscopy, demonstrating that BBB permeability changes within 2–5 min following peripheral inflammatory stimuli, with 30%–50% permeability increases occurring selectively at specific brain regions based on concurrent neural activity patterns. Critically, these changes showed remarkable spatial and temporal specificity; during cognitive demand, prefrontal BBB regions became more permeable to beneficial metabolites while simultaneously restricting inflammatory mediators.

**FIGURE 1:**
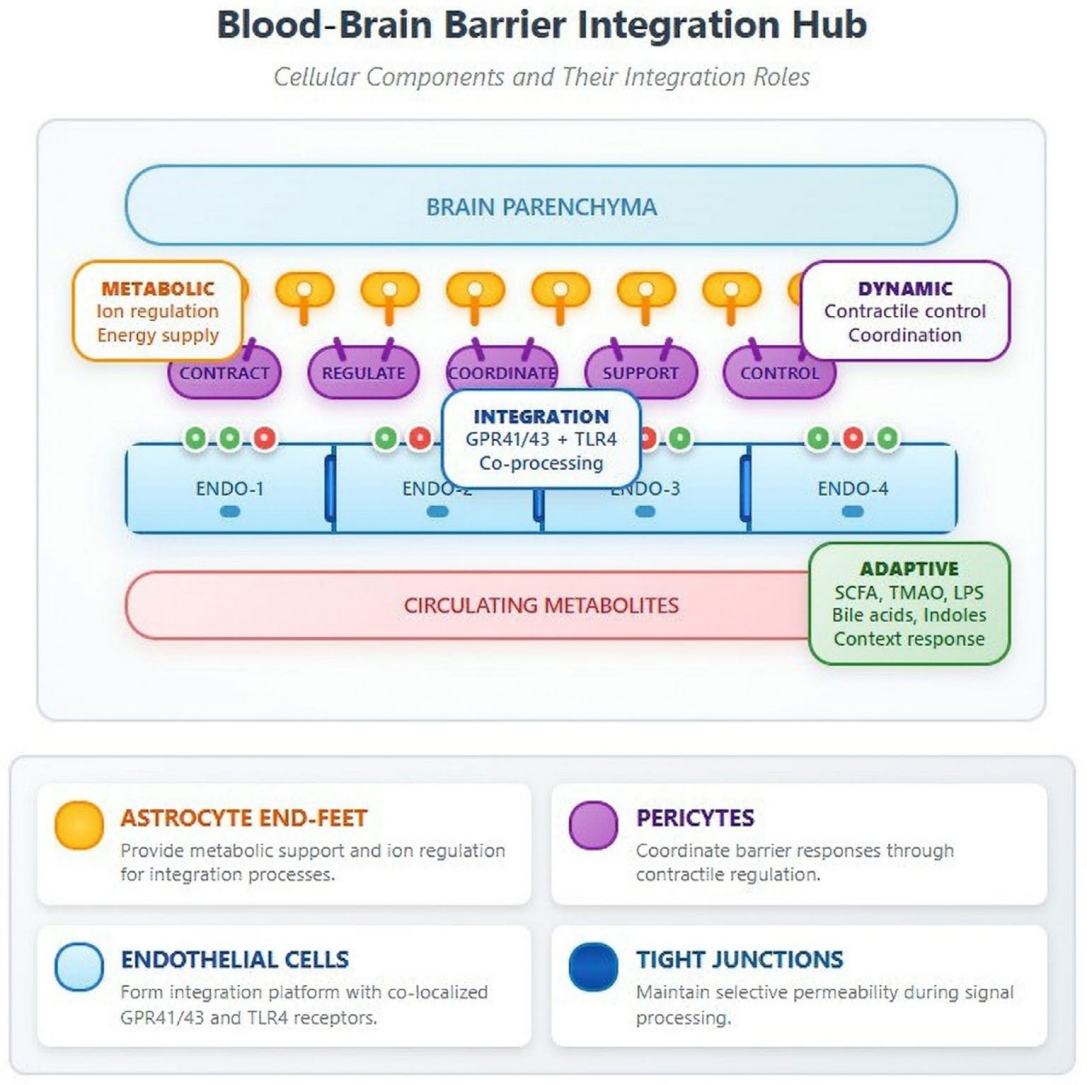
Blood–Brain Barrier Integration Hub.

Context‐dependent processing represents the hallmark of integration hub function (Figure 1). Under identical inflammatory challenges, BBB responses varied dramatically based on brain metabolic state: During enhanced neural activity, peripheral LPS produced only 10% permeability increases versus 40% during baseline states, with concurrent upregulation of efflux transporters. This adaptive response pattern demonstrates sophisticated biological integration mechanisms that adjust barrier function based on real‐time physiological assessment.

Daneman and Prat [2] revealed coordinated regulation across multiple transport systems that integrate diverse physiological streams. Glucose transporters (GLUT1), amino acid transporters (LAT1), and efflux pumps (P‐glycoprotein) showed coordinated expression changes responding to integrated signals about peripheral metabolic status, inflammatory conditions, and central energy demands. Under metabolic stress combined with cognitive demand, GLUT1 increased 60% and LAT1 showed 40% upregulation—but only when both conditions were present simultaneously; neither alone produced these coordinated changes. These mechanistic observations from human and cellular studies align with preclinical evidence, which further clarifies the temporal sequence and functional consequences of BBB–microbiota interactions.

The blood–brain barrier functions as a sophisticated molecular integration platform through coordinated interactions between astrocyte end‐feet (yellow), pericytes (purple), and endothelial cells (blue). Endothelial cells display co‐localized GPR41/43 (green) and TLR4 (red) receptors that enable simultaneous processing of diverse microbiota‐derived metabolites including beneficial short‐chain fatty acids (SCFAs), pathogenic lipopolysaccharides (LPS), trimethylamine N‐oxide (TMAO), secondary bile acids, and tryptophan metabolites such as indole derivatives. Tight junctions (dark blue bars) maintain selective permeability while allowing context‐dependent responses to this complex metabolite environment. Each endothelial cell shows distinct receptor profiles (ENDO‐1 to ENDO‐4), enabling graduated adaptive responses based on the integration of multiple simultaneous signals. Pericytes provide contractile regulation and coordinate responses across the neurovascular unit, while astrocytes supply metabolic support and structural integrity. This multicellular architecture allows the BBB to distinguish between beneficial metabolites (SCFAs, protective indoles), harmful compounds (LPS, TMAO), and context‐dependent signals (bile acids) within mixed metabolite streams, orchestrating adaptive responses that modulate barrier permeability, immune activation, and neuroprotection based on the overall molecular context and processing capacity.

### 1.2 Experimental Evidence From Animal Models

Controlled experimental studies provide mechanistic evidence for BBB‐centric pathways. Hoffman et al. [3] demonstrated age‐related changes across interconnected systems, including gut microbiome composition, brain metabolism, and vascular function in mouse models, revealing complex interactions between gut microbiota diversity and cerebrovascular health that support the BBB integration hub concept.

Recent sophisticated analyses challenge assumptions of widespread barrier breakdown in AD models. Nozohouri et al. [4] conducted a comprehensive pharmacokinetic analysis using [^13^C₁₂] sucrose as a permeability marker in Tg2576 AD mice, revealing that BBB integrity was preserved both globally and locally despite significant amyloid pathology. Despite minor tight junction disruptions near Aβ plaques observed through high‐resolution imaging, laser microdissection coupled with LC–MS/MS analysis showed no increased sucrose concentrations in regions with vascular Aβ deposition, demonstrating that structural changes do not necessarily translate to functional permeability increases.

Sweeney et al. [5] created mice with isolated BBB dysfunction through selective pericyte‐specific PDGFRβ knockout while maintaining normal gut microbiome composition. Despite identical peripheral physiology and microbiome profiles, BBB‐compromised animals developed progressive cognitive impairment across multiple domains (Morris water maze: Escape latency increased from 18 ± 3 s–34 ± 7 s, *p* < 0.001; T‐maze alternation: success decreased from 78% ± 8%–54% ± 12%, *p* < 0.01). Cognitive deficits correlated directly with BBB permeability (*r* = 0.82, *p* < 0.001), while peripheral markers showed no correlation, demonstrating causal relationships between BBB integrity and cognitive function independent of gut microbiome status. These preclinical findings converge with human imaging and biomarker studies, which provide complementary evidence for BBB‐related metabolite processing deficits in Alzheimer's disease.

### 1.3 Clinical Evidence in Humans

Individual differences in BBB integration capacity explain variability in gut–brain communication independent of microbiome composition. Recent clinical evidence demonstrates that BBB permeability is associated with different neuroinflammatory profiles in AD patients. Bruno et al. [6] investigated the relationship between BBB permeability (indicated by CSF/plasma albumin quotient) and CSF inflammatory cytokines in biologically defined AD patients. Their findings revealed that higher BBB permeability was associated with neuroprotective cytokines (IL‐4, IL‐8), while lower permeability correlated with pro‐inflammatory mediators (TNF‐α, MIP‐1β), demonstrating that the BBB actively discriminates between different inflammatory signals rather than functioning as a passive barrier.

Longitudinal human studies demonstrate that BBB dysfunction precedes classical AD pathology. Nation et al. [7] showed that individuals with early cognitive dysfunction develop brain capillary damage and BBB breakdown in the hippocampus irrespective of Alzheimer's Aβ and/or tau biomarker changes, suggesting that BBB breakdown is an early biomarker of human cognitive dysfunction independent of Aβ and tau. BBB damage predicted cognitive impairment regardless of hippocampal volume, vascular risk scores, or age, indicating that hippocampal BBB damage occurs outside normal aging trajectories. These clinical observations underscore the need for a mechanistic framework that explains how peripheral metabolite signals are interpreted at the BBB.

Together, these clinical findings highlight the importance of examining the anatomical and cellular structures that enable the BBB to interpret microbial and metabolic signals, as detailed in the following section.

### 1.4 Anatomical Foundation for Metabolite Processing

The BBB's specialized architecture enables sophisticated processing of microbiota‐derived metabolites through coordinated receptor systems positioned on the same cellular platform. Brain microvascular endothelial cells express G‐protein–coupled receptors GPR41/43 for short‐chain fatty acid detection on both luminal and abluminal surfaces, enabling monitoring of SCFAs from blood circulation and brain tissue compartments. Pattern recognition receptor TLR4 specifically detects lipopolysaccharides from gram‐negative bacteria, creating a molecular platform that simultaneously processes beneficial and pathological microbial signals.

This receptor co‐localization enables context‐dependent signal processing that distinguishes integration‐hub function. When GPR41/43 detect beneficial SCFAs while TLR4 remains inactive, coordinated signaling enhances tight junction integrity and supports neuroprotection. However, when TLR4 simultaneously detects LPS, identical SCFA inputs are processed differently with attenuated beneficial effects, demonstrating active signal integration beyond simple biochemical effects.

The BBB contains transport systems optimized for microbiota metabolite processing. Monocarboxylate transporters MCT1/MCT2 enable SCFA transport, while efflux systems including P‐glycoprotein remove harmful bacterial metabolites. Pericytes express receptors for both beneficial and pathological metabolites, enabling coordinated responses across the neurovascular unit. This transport specialization explains how intact integration capacity can selectively process beneficial microbial signals while excluding pathological ones.

Notably, Nozohouri et al. (2025) [4] demonstrated preserved global BBB function in Tg2576 mice, whereas Montagne et al. (2020) reported localized permeability increases around vascular amyloid. These findings are not contradictory: They indicate that BBB dysfunction in AD is spatially heterogeneous. Microregional deficits may selectively impair metabolite processing, e.g., pericyte signaling, endothelial transport, or receptor cross‐talk, without producing global leakage. This distinction is essential to understand region‐specific vulnerability in metabolite integration.

The subsequent sections build on this foundation by examining the BBB's functional architecture (Section 2), its progressive failure in Alzheimer's disease (Section 3), and the modulatory effects of microbiota‐derived metabolites (Section 4). Section 5 outlines bidirectional interactions between the gut and the BBB, while Sections 6 and 7 address therapeutic implications and translational priorities. Together, these sections provide an integrated framework for understanding how BBB integration capacity shapes gut–brain communication in Alzheimer's disease.

## 2 The BBB Integration Hub: Functional Architecture and Signal Processing

Here we define integration capacity as the BBB's ability to detect, prioritize, and coordinate responses to simultaneous microbial, metabolic, and inflammatory inputs.

Throughout this review, terms describing BBB “signal integration” or “coordination dynamics” refer to biological processes through which endothelial, pericytic, and astrocytic pathways evaluate and respond to multiple simultaneous microbial and inflammatory inputs.

The BBB functions as an active integration hub through sophisticated signal detection and processing mechanisms that distinguish it from simple filtration barriers. Brain microvascular endothelial cells represent biological signal processors through strategic co‐expression of diverse receptor systems on the same cellular platform, creating a molecular dashboard that simultaneously monitors multiple aspects of peripheral physiology while coordinating appropriate central responses.

### 2.1 Dynamic Signal Detection and Processing

The integration platform comprises three primary receptor classes working in coordination. Metabolite‐sensing receptors include GPR41/43 for SCFA detection, strategically positioned on both luminal and abluminal surfaces. Pathogen‐recognition receptors, particularly TLR4, serve as threat‐detection systems recognizing lipopolysaccharides from gram‐negative bacteria. Metabolic coordination receptors, including GLP‐1 receptors, enable alignment of BBB responses with overall metabolic status.

Signal convergence and cross‐talk between receptor systems form the basis of BBB integration. Rather than responding to single inputs, endothelial cells adjust their signaling output according to the combined pattern of microbial and inflammatory cues. For example, beneficial SCFA stimulation tends to support barrier stability when inflammatory pathways are inactive, whereas concurrent TLR4 activation modifies this response profile and reduces SCFA‐associated protective effects, illustrating the context‐dependent nature of BBB signal interpretation [8].

The BBB demonstrates coordinated signal‐processing mechanisms that adjust responses according to combined input patterns and physiological context. During inflammatory challenges, TLR4 signaling can override beneficial SCFA effects by shifting the hierarchy of endothelial signaling pathways [9]. Conversely, during metabolic stress, incretin‐signaling pathways receive processing priority, demonstrating hierarchical signal integration that responds to physiological demands.

Recent mechanistic work shows that hierarchical signal integration at the BBB is regulated through specific molecular interactions. NF‐κB activation downstream of TLR4 interferes with SCFA‐mediated HDAC inhibition by altering chromatin accessibility at tight‐junction gene promoters, thereby reducing the protective effects of GPR41/43 signaling [8]. Phosphoproteomic profiling of endothelial cells demonstrates distinct phosphorylation states during simultaneous GPR41/43–TLR4 activation, supporting the existence of receptor co‐activation “decision nodes” that dynamically prioritize inflammatory over metabolic inputs.

This hierarchical receptor interaction follows the cellular architecture illustrated in Figure 1, where co‐localized endothelial receptors and pericyte–astrocyte coordination provide the structural basis for dynamic signal prioritization.

A schematic representation of SCFA–GPR41/43 and LPS–TLR4 hierarchical interactions is provided in Figure S1.

### 2.2 Integration Network Coordination

Building on the receptor‐level interactions described in Section 2.1, gut–brain communication pathways provide higher‐order coordination signals that shape how the BBB integrates microbial and inflammatory inputs.

The BBB integration hub coordinates specifically with gut–brain communication pathways to optimize microbiota‐derived signal processing. The vagus nerve provides primary direct communication between gut microbiome status and BBB integration capacity, enabling real‐time coordination between peripheral microbial signals and central signal processing function [10].

Vagal afferent terminals in the gut wall express specialized receptors that directly detect microbiota‐derived metabolites and pathological signals. Short‐chain fatty acids activate GPR41/43 receptors on vagal afferents, generating signals that enhance BBB integration capacity through brainstem‐mediated pathways. Conversely, lipopolysaccharides activate TLR4 receptors on the same vagal terminals, generating competing signals that can overwhelm beneficial SCFA signaling.

The coordination between gut microbiome status, peripheral immune activation, and BBB integration represents a critical mechanism through which dysbiosis influences cognitive function. Lipopolysaccharides from dysbiotic microbiomes create specific immune activation patterns that target BBB integration infrastructure through cytokine cascades (TNF‐α, IL‐1β, IL‐6) that directly compromise endothelial cell function and pericyte viability [11]. Short‐chain fatty acids provide immune‐mediated protection through regulatory T‐cell activation producing IL‐10 and TGF‐β, cytokines that specifically protect BBB infrastructure while preserving processing capacity [8].

## 3 Integration Hub Failure in Alzheimer's Disease

Alzheimer's disease represents a systematic breakdown of BBB integration mechanisms following a predictable temporal sequence where beneficial metabolites gradually lose protective effects and may contribute to neurodegeneration when the BBB's signal‐processing capacity becomes compromised.

### 3.1 Progressive Deterioration of Signal‐Processing Mechanisms

The earliest detectable change involves integration coordinator failure through pericyte degeneration. Pericytes serve as primary coordinators of BBB integration, and their loss destabilizes the entire processing system [5, 12]. This manifests as reduced integration capacity, impaired signal discrimination, and loss of adaptive threshold control. Pericyte loss can be detected through blood‐based biomarkers years before patients show cognitive symptoms. This early decline reflects a reduction in the BBB's functional reserve, defined as the surplus physiological capacity that enables endothelial and pericyte networks to maintain selective permeability and coordinated signaling responses under metabolic or inflammatory stress. Although emerging evidence supports this framework, variation in BBB functional reserve across individuals has not yet been systematically quantified, and further studies are needed to clarify its clinical relevance.

Signal discrimination breakdown follows pericyte loss as tight junction integrity becomes progressively compromised. This represents a fundamental shift from selective, context‐dependent permeability to constitutive, uncontrolled access [13]. The BBB loses the ability to discriminate between beneficial and harmful signals, allowing previously excluded inflammatory mediators and bacterial products to reach brain tissue.

Signal‐processing mechanisms failure emerges during clinical phases as transport systems become dysfunctional. LRP1 dysfunction represents a particularly important example because it directly links integration failure to amyloid pathology [7]. As integration capacity declines, the BBB can no longer appropriately process amyloid‐β peptides for clearance, leading to progressive accumulation. Single‐cell transcriptomic analyses reveal that pericytes are highly heterogeneous across brain regions [14]. Subpopulations in the hippocampus and entorhinal cortex exhibit distinct metabolic and inflammatory profiles, suggesting that regional pericyte differences may shape local metabolite processing capacity. This may help explain why AD‐vulnerable regions show earlier loss of integration precision and reduced responsiveness to beneficial metabolites. Additional mechanistic evidence supports this concept. Phosphoproteomic profiling of brain endothelial cells shows distinct signaling states during combined SCFA and TLR4 stimulation, indicating that the BBB distinguishes inputs through hierarchical and context‐dependent pathway activation.

### 3.2 Microbiota Metabolite Processing Reserve and Individual Susceptibility

In this review, we use the term “metabolite processing reserve” to describe the BBB's remaining capacity to maintain selective metabolite handling under physiological or inflammatory stress. This is a conceptual framework rather than a formally validated biomarker, intended to help explain inter‐individual variability in BBB resilience.

The concept of BBB microbiota metabolite processing reserve provides a framework for understanding individual differences in gut‐brain communication effectiveness and AD susceptibility patterns. Processing reserve represents the BBB's total capacity for handling complex microbiota‐derived signal coordination challenges, specifically the ability to simultaneously process beneficial metabolites (SCFAs) while managing pathological signals (LPS, TMAO) [3]. Although most evidence centers on pericytes, endothelial tight‐junction signaling and astrocytic metabolic support also influence BBB integration capacity.

Individuals with high BBB metabolite processing reserve can maintain appropriate SCFA utilization and pathological metabolite exclusion even when challenged by significant gut dysbiosis. This reserve capacity explains why some individuals remain cognitively healthy despite substantial alterations in gut microbiome composition or elevated levels of harmful bacterial metabolites [15]. Conversely, individuals with low metabolite processing reserve may develop cognitive symptoms with relatively mild gut dysbiosis because their BBB cannot efficiently discriminate between beneficial and harmful microbial signals. Recent mechanistic studies clarify how this discrimination occurs: SCFAs enhance tight‐junction stability through GPR41/43‐mediated HDAC inhibition, while LPS activates TLR4–NF‐κB pathways that disrupt junctional integrity and promote endothelial stress. These opposing signaling routes provide a molecular basis for BBB signal discrimination without implying computational properties [8].

Because this concept is still preliminary, additional studies are needed to determine how metabolite processing reserve varies across populations and disease stages.

## 4 Metabolite‐Mediated Modulation of BBB Integration Capacity

### 4.1 Integration‐Enhancing Metabolites: SCFAs as Capacity Modulators

Short‐chain fatty acids represent the most extensively studied example of how gut‐derived metabolites modulate BBB signal processing capacity [16, 17]. SCFAs function as processing capacity enhancers through coordinated mechanisms that strengthen the BBB's computational infrastructure. Recent analyses confirm that SCFAs, particularly butyrate and propionate, serve as key mediators linking beneficial gut bacteria to neuroprotective outcomes through HDAC inhibition, GPR41/43 activation, and anti‐inflammatory cytokine production [18, 19].

Butyrate crosses the BBB through monocarboxylate transporters MCT1 and MCT2, functioning as an HDAC inhibitor leading to increased transcription of tight junction proteins including *claudin‐5*, *occludin*, and ZO‐1 [8, 16]. This transcriptional enhancement occurs specifically in response to processing demands, demonstrating context‐dependent capacity modulation.

Germ‐free mouse studies support SCFAs as capacity modulators. Braniste et al. [16] demonstrated that germ‐free mice exhibit decreased tight junction protein expression and increased BBB permeability. Butyrate administration restored both tight junction protein expression and barrier selectivity within 72 h. Some studies also report context‐dependent or limited effects of SCFAs on BBB properties, indicating that their influence may vary depending on metabolic or inflammatory conditions [18].

### 4.2 Integration‐Disrupting Metabolites: LPS And TMAO as Drivers of Signal‐Processing Failure

Pathological metabolites overwhelm and damage BBB signal‐processing pathways through distinct mechanisms. Lipopolysaccharides specifically target BBB structural and signaling architecture through TLR4 receptors on brain endothelial cells [20, 21]. TLR4 activation initiates NF‐κB signaling cascades that disrupt tight junction proteins while impairing coordinated signaling between endothelial cells, pericytes, and astrocytes that enable processing function.

Banks and Robinson [22] demonstrated that even high‐dose peripheral LPS shows minimal direct BBB penetration yet produces profound central effects through computational system disruption. Low‐dose chronic LPS exposure induces progressive processing overload without causing acute toxicity, consistent with the integration‐hub model. Trimethylamine N‐oxide represents another critical example of metabolite‐mediated integration disruption. TMAO, produced by gut microbiota from dietary choline, carnitine, and betaine, may cross the blood–brain barrier and has been associated with changes in neural and endothelial physiology [23]. Large‐scale studies report that cerebrospinal fluid TMAO levels tend to be higher in individuals with mild cognitive impairment and AD, with potential correlations to AD‐related biomarkers [23, 24, 25].

TMAO may contribute to reduced BBB integration capacity through mechanisms distinct from those associated with LPS exposure. TMAO promotes neuroinflammation through microglial activation and increases oxidative stress in brain endothelial cells [24]. Recent experimental studies have reported that TMAO administration is associated with cognitive changes and reductions in BBB‐related proteins such as ZO‐1, occludin, and PDGFRβ [25]. Recent clinical work further strengthens this association, demonstrating that elevated plasma and CSF TMAO levels correspond to region‐specific increases in BBB permeability in early‐stage AD, particularly within the hippocampal and parietal microvascular networks [26]. The study also reported TMAO‐induced downregulation of tight‐junction proteins and pericyte‐signaling pathways as key drivers of BBB vulnerability. Notably, these effects were observed independently of vascular risk factors, supporting TMAO as a direct modulator of BBB integrity in humans.

Secondary bile acids represent another important class of microbiota‐derived metabolites that demonstrate context‐dependent effects on BBB function. Deoxycholic acid and lithocholic acid, produced by bacterial species including *Clostridium scindens* and *Eggerthella lenta* , can modulate BBB permeability through Rac1‐dependent tight junction disruption at high concentrations while showing protective effects at lower doses [27, 28]. These metabolites activate receptors including TGR5 and FXR, leading to dose‐dependent outcomes that depend on BBB integration capacity [29].

Tryptophan metabolites, particularly indole derivatives produced by gut bacteria, demonstrate neuroprotective effects through aryl hydrocarbon receptor (AhR) activation. Indole‐3‐propionic acid from *Clostridium sporogenes* and indole‐3‐aldehyde from *Lactobacillus reuteri* enhance BBB integrity, reduce oxidative stress, and promote microglial M2 polarization [30, 31]. These metabolites support adult neurogenesis and provide anti‐inflammatory effects that protect BBB integration capacity during challenging conditions [32, 33].

A summary of the major microbiota‐derived metabolites, their BBB targets, and functional consequences is provided in Table 1.

**TABLE 1:** Microbiota‐derived metabolites and their effects on BBB integration function.

Metabolite	Microbial source	BBB receptors/targets	Molecular effects	Functional outcome	Key references
Short‐chain fatty acids (SCFAs)	Butyrate: *Faecalibacterium prausnitzii* , *Clostridium butyricum* Propionate: bacteroides spp., veillonella spp. Acetate: Bifidobacterium spp., lactobacillus spp.	GPR41/43 MCT1/ MCT2 (transporters)	• HDAC inhibition → ↑ claudin‐5, occludin, ZO‐1 • ↑ Anti‐inflammatory IL‐10, TGF‐β • ↓ NF‐κB activation • ↑ Pericyte survival and function • Enhanced mitochondrial metabolism	Integration enhancement: • Improved barrier selectivity • Enhanced signal processing capacity •Neuroprotection • Maintained cognitive function • Reduced neuroinflammation	[8, 16, 17, 18, 19]
Lipopolysaccharides (LPS)	Gram‐negative bacteria: *Escherichia coli* , *Bacteroides fragilis* , *Prevotella copri* , Enterobacter spp.	TLR4 CD14 MD‐2	• TLR4/MyD88 pathway activation • ↑ NF‐κB → TNF‐α, IL‐1β, IL‐6 • ↓ Tight junction proteins • ↑ Matrix metalloproteinases • Pericyte degeneration	Integration disruption: • Increased barrier permeability • Overload of BBB signal‐processing pathways •Neuroinflammation • Cognitive impairment • Accelerated neurodegeneration	[11, 20, 21]
Trimethylamine N‐oxide (TMAO)	Choline metabolism: Escherichia/shigella, *klebsiella pneumoniae* , *enterobacter aerogenes* Carnitine metabolism: Various gut bacteria → TMA → hepatic FMO3 → TMAO	Multiple targets (no specific receptor identified)	• ↑ Oxidative stress (ROS generation) • ↑ Microglial activation • ↓ ZO‐1, occludin, PDGFRβ • Endothelial dysfunction • ↑ Neuroinflammatory cascades	Progressive dysfunction: • Gradual barrier breakdown • Impaired signal discrimination • Cognitive decline • Increased AD pathology • Accelerated aging	[23, 24, 25, 26]
Secondary bile acids	Deoxycholic acid: *Clostridium scindens* , *clostridium hiranonis* Lithocholic acid: *Eggerthella lenta* , *clostridium sordellii*	TGR5 FXR PXR	• TGR5 → cAMP → anti‐inflammatory • FXR → metabolic regulation • High concentrations → cytotoxicity • Context‐dependent effects • Rac1 activation → occludin phosphorylation • BBB permeabilization via tight junction disruption	Dose‐dependent: • Low doses: protective • High doses: toxic • Modulation of BBB permeability • Variable cognitive effects • Integration capacity dependent	[27, 28, 29]
Tryptophan metabolites	Indole‐3‐propionic acid: *Clostridium sporogenes* Indole‐3‐aldehyde: *Lactobacillus reuteri* Indole‐3‐acetic acid: Various clostridium spp. Indole: *Escherichia coli* , Bacteroides spp.	AhR (Aryl hydrocarbon receptor)	• AhR activation → neuroprotection • ↑ Antioxidant response • ↑ Tight junction integrity • Microglial M2 polarization • VEGF‐α upregulation • Adult neurogenesis promotion	neuroprotective: • Enhanced BBB integrity • Reduced oxidative stress • Anti‐inflammatory effects • Improved cognitive function • Delayed neurodegeneration • Neurogenesis support	[30, 31, 32, 33]

*Note:* This table summarizes the major classes of microbiota‐derived metabolites, their bacterial sources, BBB molecular targets, and functional outcomes. Short‐chain fatty acids (SCFAs) enhance integration capacity through multiple coordinated mechanisms including HDAC inhibition and anti‐inflammatory pathways. Pathological metabolites like LPS and TMAO impair BBB signal‐processing pathways through distinct mechanisms, leading to barrier dysfunction and cognitive impairment. Secondary bile acids show context‐dependent effects, with deoxycholic and lithocholic acids demonstrated to increase BBB permeability through Rac1‐dependent tight junction disruption at high concentrations, while showing protective effects at lower doses. Tryptophan metabolites, particularly indole derivatives, demonstrate neuroprotective effects through AhR‐mediated mechanisms, including enhanced BBB integrity and promotion of adult neurogenesis. The integration hub model explains why identical metabolite concentrations can produce different cognitive outcomes based on the BBB's ability to process complex signal environments. Note: Effects are context‐dependent and influenced by BBB integration capacity, concurrent signal environment, and individual metabolite processing reserve. Beneficial metabolites may become ineffective or pathological when BBB signal‐processing capacity is compromised. Overload of BBB signal‐processing pathways refers to reduced capacity of the BBB to prioritize or integrate complex metabolite mixtures, leading to loss of selective barrier function.

### 4.3 Complex Metabolite Pattern Processing

The integration hub model predicts that BBB function depends not on individual metabolite levels but on the capacity to process complex, mixed signal environments typical of real physiological conditions. When beneficial metabolites like SCFAs and tryptophan derivatives are present alongside inflammatory signals like LPS, the BBB can coordinate and prioritize these competing inputs. Individuals with intact integration capacity can maintain beneficial signal processing even in mixed environments, while those with compromised capacity experience processing overload and impaired signal discrimination.

This complex metabolite processing capacity explains the documented variability in clinical responses to microbiome interventions. Patients with preserved BBB integration function show consistent positive responses to probiotic therapies that increase beneficial metabolite production. However, patients with compromised integration capacity may show minimal responses or even adverse outcomes, as their BBB cannot effectively discriminate between beneficial and pathological signals in complex metabolite environments. Importantly, these metabolites do not act independently: their effects on BBB function arise from converging and competing signaling pathways, making the overall response dependent on the combined metabolite pattern rather than on individual molecules.

These metabolite‐dependent shifts in BBB function provide the basis for understanding how barrier physiology interacts bidirectionally with gut‐derived signals, as discussed in the following section.

## 5 Bidirectional BBB‐Gut Integration: Dynamic System Interactions

Evidence for bidirectional BBB–gut interactions is strongest in animal models, while human studies primarily support gut‐to‐BBB influences and provide only preliminary indications of BBB‐to‐gut feedback. The relationship between BBB dysfunction and gut dysbiosis appears fundamentally bidirectional instead of representing a simple linear cascade [34, 35]. Gut dysbiosis can drive BBB dysfunction through chronic exposure to lipopolysaccharides that directly disrupt tight junction proteins through TLR4‐mediated inflammatory cascades, while reduced SCFA production removes protective signals that normally support BBB integrity.

Conversely, BBB dysfunction can promote gut dysbiosis through mechanisms that affect vagal nerve signaling patterns, influencing gut motility, secretion patterns, and microbial selection pressures [10]. Altered immune responses secondary to BBB dysfunction can change the gut's inflammatory environment in ways that favor pathogenic bacterial overgrowth while suppressing beneficial species.

At the molecular level, BBB and gut dysfunction interact through shared inflammatory pathways and metabolic systems [35, 36]. Chronic production of pro‐inflammatory cytokines from dysbiotic gut bacteria can directly damage pericytes and endothelial cells, creating a positive feedback loop where BBB dysfunction becomes progressively more severe. Simultaneously, impaired BBB clearance mechanisms allow accumulation of gut‐derived toxins that would normally be efficiently removed from brain tissue. The bidirectional nature of BBB‐gut interactions creates individual patterns of dysfunction that require personalized therapeutic approaches. Table 2 illustrates the critical differences between intact and compromised BBB integration capacity across multiple functional parameters. While gut‐to‐BBB signaling is supported by both human and experimental evidence, BBB‐to‐gut feedback remains preliminary and should be interpreted as a working hypothesis requiring further validation. The table summarizes conceptual patterns supported by experimental and clinical evidence, but several entries represent integrative interpretations rather than direct one‐to‐one empirical measurements. In this table, entries related to tight‐junction proteins, transporter regulation, permeability shifts, and cytokine profiles derive from direct experimental or clinical evidence, whereas comparative assessments of “processing capacity,” “signal discrimination,” or broader integration patterns represent conceptual synthesis based on integrated findings.

**TABLE 2:** Comparison of intact versus compromised BBB integration capacity.

Integration parameter	Intact BBB function	Compromised BBB function	Clinical consequences	Key references
SCFA processing capacity	• Efficient GPR41/43 signaling • Robust HDAC inhibition • Enhanced tight junction expression • Anti‐inflammatory response	• Reduced receptor sensitivity • Impaired HDAC pathway • Decreased tight junction proteins • Attenuated beneficial effects	Intact: Cognitive protection despite gut challenges Compromised: SCFA supplementation ineffective, cognitive decline despite “healthy” microbiome	[8, 16, 17]
LPS response management	• Controlled TLR4 activation • Rapid inflammatory resolution • Maintained barrier selectivity • Effective efflux mechanisms	• TLR4 hyperactivation • Sustained neuroinflammation • Uncontrolled permeability • Impaired clearance systems	Intact: Resilience to gut dysbiosis, maintained cognitive function Compromised: Accelerated neurodegeneration, high sensitivity to gut inflammation	[11, 20, 21, 22]
Signal integration processes	• Context‐dependent processing • Temporal signal coordination • Appropriate threshold modulation • Efficient cross‐talk	• Signal overload • Loss of temporal discrimination • Threshold dysregulation • Impaired signal discrimination	Intact: Optimal intervention responses, personalized therapeutic success Compromised: Unpredictable treatment responses, intervention failures	[1, 2, 7]
Pericyte coordination	• Normal PDGFRβ expression • Stable neurovascular coupling • Coordinated barrier responses • Preserved contractile function	• Pericyte degeneration • Loss of neurovascular coupling • Uncoordinated responses • Reduced contractile capacity	Intact: Preserved cognitive reserve, delayed AD onset Compromised: Early cognitive decline, accelerated AD progression	[5, 7, 12, 13]
Transport system function	• Coordinated transporter expression • Efficient SCFA uptake (MCT1/2) • Effective efflux systems • Selective permeability	• Dysregulated transporter expression • Impaired beneficial metabolite uptake • Reduced efflux capacity • Compromised selectivity	Intact: Effective metabolite utilization, therapeutic responsiveness Compromised: Metabolite accumulation, drug resistance, therapeutic failure	[16, 17]
Response to interventions	• Consistent therapeutic responses • Effective probiotic interventions • Lifestyle modification benefits • Predictable outcomes	• Variable/poor responses • Probiotic intervention failures • Limited lifestyle benefits • Unpredictable outcomes	Intact: High therapeutic success, precision medicine effectiveness Compromised: Treatment resistance, need for BBB‐targeted interventions first	[7, 15, 36]

*Note:* This table illustrates the functional differences between preserved and impaired BBB integration systems across key parameters that determine metabolite‐processing effectiveness in Alzheimer's disease. The comparison demonstrates how BBB integration capacity directly impacts clinical outcomes, explaining individual variability in gut–brain communication despite similar peripheral conditions. Intact BBB function is characterized by efficient signal processing, maintained selectivity, and preserved therapeutic responsiveness, while compromised function shows reductions across all functional domains. These differences explain why individuals with identical gut microbiomes can exhibit vastly different cognitive trajectories and intervention responses. The framework supports the integration hub model by demonstrating that BBB function exists on a spectrum of capacity rather than simple intact/disrupted states, with functional capacity determining resilience to metabolite challenges and response to interventions. Biomarker measurements such as sPDGFRβ levels offer potential clinical tools for assessing BBB integrity, while functional assessments of signal processing capacity may predict therapeutic outcomes. This table is intended as a conceptual synthesis of patterns described in the literature and should not be interpreted as a quantitative or empirical comparison.

## 6 Therapeutic Implications: Targeting Integration Hub Function

The integration hub model fundamentally reframes therapeutic approaches to AD prevention and treatment. Instead of focusing solely on reducing peripheral pathology or optimizing gut health, this framework suggests that interventions may benefit from supporting BBB integration capacity while also addressing the peripheral signals processed by this system.

### 6.1 Microbiota Metabolite Processing Restoration as Primary Target

Therapeutic strategies may aim to support the BBB's capacity to appropriately process microbiota‐derived metabolites instead of simply optimizing gut microbiome composition. This approach recognizes that even beneficial microbiomes cannot support cognitive health when BBB metabolite processing systems are compromised [5].

Interventions that specifically enhance the BBB's ability to detect and utilize short‐chain fatty acids represent promising therapeutic approaches. Compounds that upregulate GPR41/43 receptor expression or enhance HDAC inhibition pathways have been associated with improved butyrate responsiveness even in the presence of concurrent inflammatory signals [8]. Exercise interventions have been associated with improvements in SCFA‐related processing capacity through the promotion of endothelial health and pericyte function.

Since pericytes serve as critical coordinators of BBB metabolite processing, interventions that specifically protect pericyte function during gut dysbiosis represent potential therapeutic targets. Compounds that enhance PDGF‐β signaling or provide metabolic support for pericyte energy demands can maintain processing capacity even during inflammatory challenges [5]. Because targeted BBB‐directed therapies remain largely experimental, early human studies are still limited and further validation is required before these approaches can be reliably applied in clinical settings.

### 6.2 Personalized Medicine Approaches

The complexity of BBB‐dysbiosis interactions necessitates personalized assessment and intervention strategies that account for individual patterns of dysfunction [37, 38]. Combined evaluation of gut microbiome composition, metabolite profiles, and BBB integrity markers can identify which pathways predominate in individual patients and guide targeted intervention selection [12, 36].

Sequential intervention protocols may involve initial restoration of endothelial health, through exercise, metabolic support, or SCFA‐enhancing strategies, followed by targeted modulation of inflammatory pathways once BBB responsiveness has improved. This approach differs from simultaneous interventions by prioritizing recovery of processing capacity before addressing secondary signaling abnormalities [7, 13].

Despite these opportunities, translating microbiota‐ and BBB‐targeted strategies into clinical practice remains challenging. Targeted delivery of GPR41/43 enhancers or TLR4 antagonists to brain endothelium is limited by narrow therapeutic windows and the risk of interfering with physiological immune surveillance. Preliminary human data remain limited: small pilot trials exploring SCFA‐enhancing diets or probiotics targeting inflammatory pathways have shown modest effects on endothelial biomarkers but inconsistent cognitive outcomes, underscoring the need for rigorous studies in AD populations [39, 40, 41]. Although these BBB‐targeted strategies remain exploratory, this does not diminish the relevance of the integration hub framework; rather, it underscores the need for improved delivery approaches optimized for BBB signaling.

## 7 Clinical Translation and Research Priorities

Translation of the BBB integration hub model into clinical practice requires the development of practical assessment tools and targeted therapeutic approaches. Current blood‐based BBB markers such as sPDGFRβ and albumin ratios can be combined with microbiome analysis and metabolomic profiling to create comprehensive risk assessment platforms [7, 12].

The integration hub model suggests specific pharmaceutical development priorities focusing on compounds that enhance BBB metabolite processing capacity instead of targeting individual pathways in isolation. Promising approaches include GPR41/43 signaling enhancers that amplify SCFA responsiveness, selective TLR4 antagonists that reduce LPS sensitivity while preserving beneficial immune functions, and combination therapeutics that simultaneously optimize microbial metabolite production and BBB processing capacity [8, 20].

Recent systematic reviews have identified critical knowledge gaps requiring urgent research attention [42, 43]. Priority areas include characterizing temporal relationships between BBB and gut dysfunction across diverse populations, developing validated assessment protocols that capture both systems simultaneously, and creating personalized intervention strategies based on individual BBB metabolite processing capacity. Standardized gut–brain assessment protocols may include a combined evaluation of CSF/plasma albumin ratio, circulating SCFA and TMAO levels, sPDGFRβ as a marker of pericyte injury, and cognitive endpoints such as delayed recall or processing speed, allowing objective measurement of BBB–microbiome interactions.

Initial pilot studies, including SCFA‐supplementation trials and TMAO‐reduction interventions in individuals with mild cognitive impairment, provide preliminary models for integrating metabolic, microbiome, and BBB biomarkers within a unified clinical framework. Emerging research has highlighted the need for standardized protocols to assess gut–brain axis function, biomarker validation studies for clinical translation, and development of combination therapeutic approaches that address both peripheral and central components of the axis [18, 19].

## 8 Conclusion

The evidence demonstrates that both BBB dysfunction and gut dysbiosis represent critical pathways linking peripheral health to cognitive decline in Alzheimer's disease [5, 7, 44]. Instead of competing hypotheses, these mechanisms interact synergistically through bidirectional pathways that create complex but predictable patterns of pathophysiology [34, 35].

BBB dysfunction provides a measurable interface where peripheral signals are processed and can become pathological when the barrier's capacity is compromised [12, 13]. This system offers concrete biomarkers for early detection and specific targets for therapeutic intervention. Gut dysbiosis generates harmful peripheral signals that can overwhelm BBB processing capacity while simultaneously reducing beneficial signals that support barrier function [8, 45].

The clinical implications extend beyond academic interest to provide practical guidance for prevention and treatment strategies [15, 36]. Individual patients may require assessment of both systems to determine optimal intervention approaches. Early intervention focusing on either system may prevent decline in the other, while advanced disease may require simultaneous targeting of both pathways [37].

This integrative perspective helps organize current evidence across BBB and gut pathways without implying replacement of existing mechanistic models. This integrated model moves beyond simple linear explanations to embrace the complexity of biological systems while maintaining focus on measurable and modifiable factors [9, 10]. Future research should prioritize characterizing temporal relationships between BBB and gut dysfunction across diverse populations, developing validated assessment tools that capture both systems simultaneously, and creating personalized intervention protocols that address individual patterns of pathophysiology [38]. However, most available evidence remains associative, and causal relationships between BBB–microbiota interactions and human cognitive decline have not yet been established, underscoring the need for longitudinal and interventional studies.

Success in these endeavors may finally provide the mechanistic understanding necessary for effective prevention and treatment of Alzheimer's disease.

## Conflicts of Interest

The authors declare no conflicts of interest.

## Supporting information


**Figure S1:** Hierarchical integration of SCFA–GPR41/43 and LPS–TLR4 signaling at the blood–brain barrier. This schematic illustrates how endothelial cells integrate beneficial short‐chain fatty acid (SCFA) signals via GPR41/43 with pro‐inflammatory cues mediated by TLR4 activation. Under concurrent stimulation, TLR4‐driven NF‐κB activation suppresses SCFA‐mediated HDAC inhibition and reduces transcription of tight‐junction proteins, resulting in diminished barrier‐reinforcing effects. The diagram highlights how hierarchical pathway interactions shape BBB responses to complex microbial‐metabolite environments.

## Data Availability

Data sharing not applicable to this article as no datasets were generated or analysed during the current study.
