# Supplementary figures and images for: The Blood–Brain Barrier as an Integration Hub in Alzheimer's Disease: How Microbiota Metabolites Modulate Central Signal Processing
Source: CNS Neurosci Ther. 2025 Dec 14;31(12):e70703. doi: 10.1002/cns.70703 (PMC12703014; doi:10.1002/cns.70703)

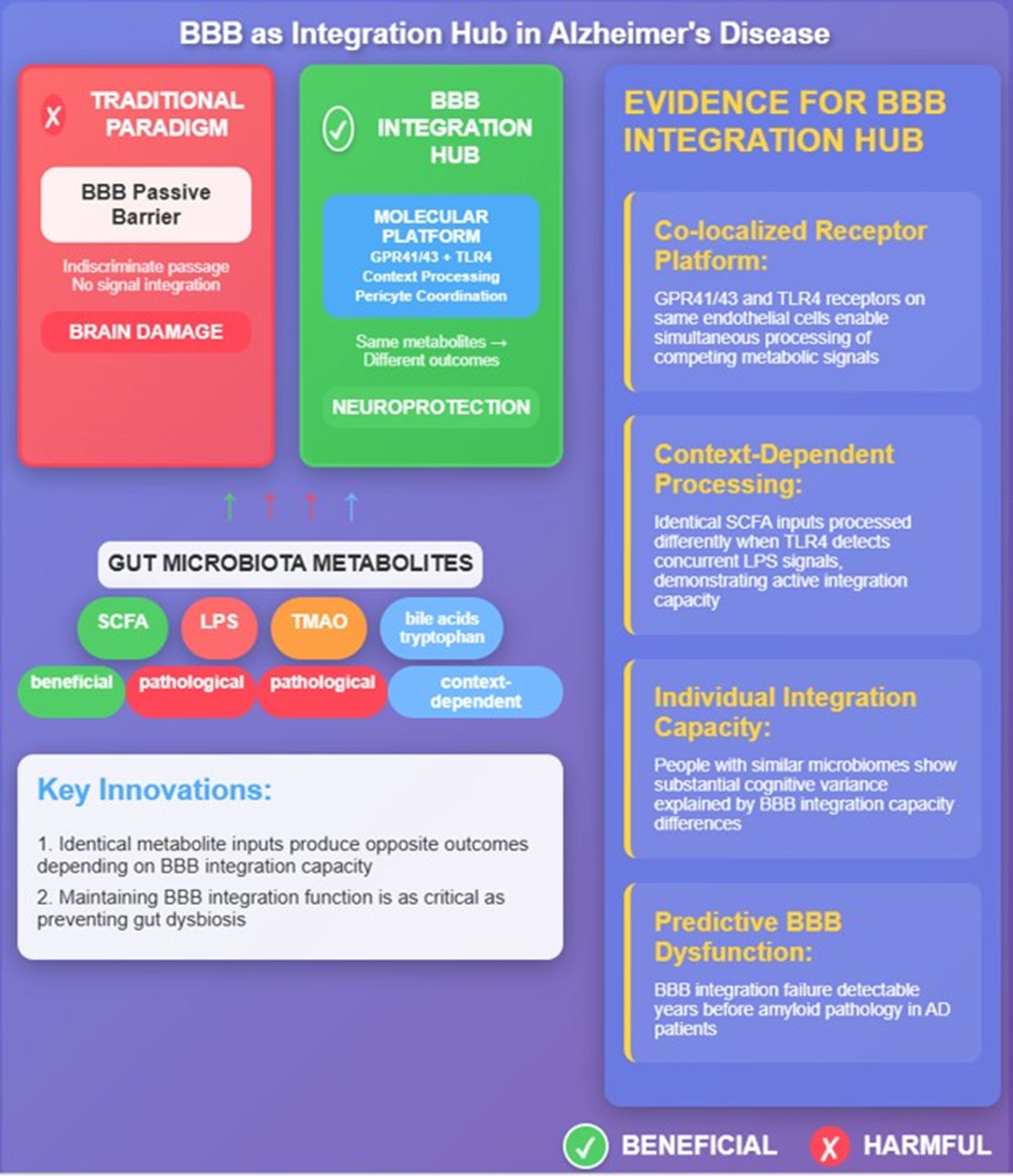

Supplement: Supplementary file 1 — Figure S1: Hierarchical integration of SCFA–GPR41/43 and LPS–TLR4 signaling at the blood–brain barrier. This schematic illustrates how endothelial cells integrate beneficial short‐chain fatty acid (SCFA) signals via GPR41/43 with pro‐inflammatory cues mediated by TLR4 activation. Under concurrent stimulation, TLR4‐driven NF‐κB activation suppresses SCFA‐mediated HDAC inhibition and reduces transcription of tight‐junction proteins, resulting in diminished barrier‐reinforcing effects. The diagram highlights how hierarchical pathway interactions shape BBB responses to complex microbial‐metabolite environments. [file CNS-31-e70703-s001.jpg]
